# Dissipative Particle Dynamics: Simulation of Chitosan–Citral Microcapsules

**DOI:** 10.3390/polym17050678

**Published:** 2025-03-03

**Authors:** Wensheng Wu, Zhiwei Li, Dachun Feng, Qing Tang, Shuijiao Liu, Wenjing Lin

**Affiliations:** 1 Guangdong Provincial Key Laboratory of Environmental Health and Land Resource, School of Environmental and Chemical Engineering, Zhaoqing University, Zhaoqing 526061, China; 2004010015@zqu.edu.cn (W.W.); tangqing2013@126.com (Q.T.); liushuijiao2016@126.com (S.L.); 2 College of Information Science and Technology, Zhongkai University of Agriculture and Engineering, Guangzhou 510225, China; fdczhku@sina.com; 3 School of Chemical Engineering and Light Industry, Guangdong University of Technology, Guangzhou 510006, China

**Keywords:** dissipative particle dynamics (DPD) simulation, microcapsules, chitosan

## Abstract

In this paper, the dissipative particle dynamics (DPD) method is used to simulate the self-assembly process, appearance, mesoscopic structure, and wrapping properties of microcapsules formed with citral as the core material and chitosan and sodium alginate as the single-wall materials, and with citral as the core material and chitosan-sodium alginate, chitosan–methylcellulose, sodium alginate–chitosan, and sodium alginate–methylcellulose as the double-wall materials. The effects of chitosan content and wall material composition on the structure, morphology, encapsulation performance, and stability of microcapsules are compared and analyzed. In addition, the microcapsules are deeply analyzed by using the mesoscopic structure, radial distribution function, and diffusion coefficient. This study provides a new idea and method for the preparation of citral microcapsules, and is of great significance for the design and development of new composite wall microcapsules.

## 1. Introduction

Microencapsulation is an important technology for making functional materials, which are used for packaging and storing trace substances. Microcapsule technology plays an important role in drug encapsulation [1,2,3,4,5,6,7], pesticide encapsulation [8,9,10,11], food encapsulation [12], and environmental pollution treatment [13,14]. In microcapsule technology, wall materials that can be used as shell materials are generally natural, semisynthetic or fully synthetic polymer materials. Coated materials are called core materials, which can be compounds or mixtures in powder, liquid, gas, or solid states. During the preparation of microcapsules, the types of wall and core materials, the volume fraction of each component and the pH value of the system all affect the synthesis, storage, and release properties of microcapsules. Especially with regard to drug loading and release, it is necessary to consider the influence of wall materials and components on the synthesis, storage, and release properties of microcapsules when the required drugs are packaged and transported to the diseased site for treatment.

Chitosan (Cts) [15], sodium alginate (Alg) [16], and methyl cellulose (MC) [17,18] are excellent wall materials in microcapsule technology due to their advantages of wide sources, good film-forming properties, stable performance, safety, nontoxicity, green environmental protection, and biodegradability.

Essential oil has a wide range of uses. For example, it can help skin moisturize and replenish water, promote blood circulation, relieve body discomfort, aid anti-inflammation and sterilization, and help restore hair luster. Citral is an essential oil with strong physiological activities, such as anticancer [19], antimutagenic [20], anti-inflammatory [21], antioxidant [22], antiviral [23], and antibacterial activities [24]. Essential oils are highly volatile and need to be sealed for storage. Microencapsulation is a common method for addressing this issue. To optimize encapsulation performance, it is critical to understand the self-assembly dynamics between core and wall materials at the mesoscopic scale. Dissipative particle dynamic (DPD) simulation provides a powerful tool to visualize such processes [25,26]. Therefore, essential oil is often used as the core material in microencapsulation technology.

Dissipative particle dynamic (DPD) simulation [25,26] is a commonly used research method in the field of chemistry that can not only solve the problem that some experimental techniques cannot, but can also greatly reduce experimental time and cost. This simulation technique can provide a convenient and intuitive way to observe the morphological changes in microcapsules and study their properties. DPD simulation can be used to analyze the aggregation process of the core material wrapped by the wall material and to study the encapsulation performance of the microcapsules. In recent years, DPD simulation has attracted great attention in microencapsulation applications, such as nanofluids [27], drug delivery [28], water cluster structures [29], polymer self-assembly [30], surface chemistry [31,32,33], ionic liquid microemulsion [34], phase diagram [35,36], and environmental protection [37].

In this paper, a DPD simulation strategy is used to study the formation process of Cts–Citral microcapsules, with Cts as the main wall material, Alg and MC as the auxiliary wall materials, and citral as the core material. First, the aggregation formation process and influencing factors of single-walled material microcapsules formed by Cts-coated citral are studied. Then, based on the single-wall material microcapsule, Alg and MC are added to replace part of the Cts. The effects of Alg and MC on the formation process of Cts–Citral microcapsules are studied by adding auxiliary wall materials and comparing the encapsulation performance of Cts–Citral microcapsules with single-wall materials. This study can provide a theoretical reference for the preparation of Cts–Citral microcapsules and the optimization of encapsulation performance.

## 2. Method

In this study, the DPD method is employed to simulate the self-assembly process of microcapsules. The DPD parameters and interaction forces are set according to the established protocols, as described in previous studies [25,26].

### 2.1. Coarse-Grained Model

**Figure 1:**
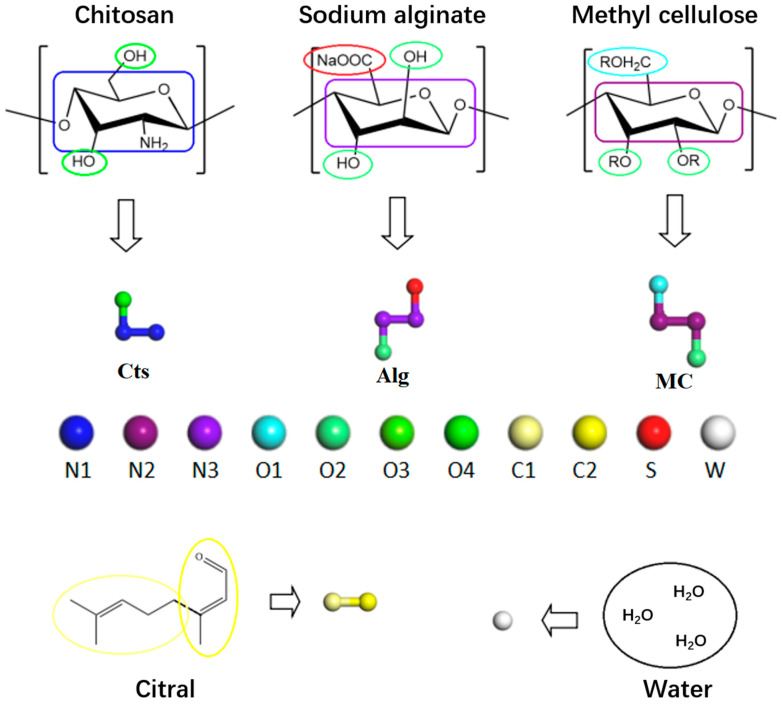
Molecular structures and coarse-grained models.

The simulated wall materials are Cts, Alg, and MC, and the core material is citral. The simulated environment is a neutral environment rich in water molecules. The coarsening treatment of molecules divides the molecules into different beads, and each bead represents one or more atomic clusters [38]. Using the coarse-grained beads method, the molecular structure formula of each substance and the corresponding coarse-grained model are shown in Figure 1. According to the molecular structure and the size of the beads, the molecules are divided as follows: one Alg molecule is composed of two N3 beads, one S bead, and one O3 bead; the MC molecule is composed of two N2 beads, one O1 bead, and one O2 bead; the Cts molecule is composed of two N1 beads and one O4 bead; the citral molecule is composed of one C1 bead and one C2 bead; and the three water molecules are composed of one W bead. In this simulation experiment, the molecular polymerization degree is set as 20.

### 2.2. Interaction Parameters Between Beads

After the coarse-grained model is determined, the interaction parameters between each pair of beads can be calculated by introducing the bead repulsion parameter *a_ii_* or *a_i__j_* and Flory Huggins parameter *x_ij_* [39].

The mutual exclusion parameter is obtained using the following formula:

*a_ii_* = 75*k_B_T/ρ* (1)

where *a_ii_* is the repulsion parameter between the same types of particles. The compressibility of pure fluid can be set as *ρ* = 3 (close to that of water), and the value of *k_B_T* = 1 can be used in the simulations. The values of the repulsion parameters between different types of particles (*a_ij_*) are linearly related to the Flory–Huggins parameters (*x_ij_*) according to the equation [38]:

*a_ij_* = *a_ii_* + 3.27*x_ij_* (2)

For the two different components *i* and *j*, the Flory Huggins parameter *x_ij_* is calculated via the following formula:

(3) $$x_{ij}=\frac{\Delta E^{mix}V_{r}}{RT\varphi_{i}\varphi_{j}V}$$

where *R* is the ideal gas constant, *T* is the absolute temperature, *V* is the total volume of the mixed component system, *V*_r_ is the relative volume, and *φ*_i_ and *φ*_j_ are the volume fractions of components *i* and *j*, respectively. Δ*E*^mix^ is the mixing energy, which can be calculated using the following formula:

(4) $$\Delta E^{mix}=E_{ij}-(E_{i}+E_{j})$$

where *E_ij_* is the total potential energy of components *i* and *j* and *E_i_* and *E_j_* are the potential energy of pure components *i* and *j*, respectively. The box size of the simulation system is 200 × 200 × 200 Å^3^. The COMPASS force field is used in the calculation of the interaction parameters, which is carried out in the Amorphous Cell and Forcite modules of Materials Studio 7.0 software [40]. The interaction parameters between the beads are shown in Table 1.

It should be noted that N2 and N3 are the same beads, and O2, O3, and O4 are the same beads. In the structure of MC, there are two -OR groups in the branched chain, where R can be hydrogen-H or methyl-CH_3_. To make the simulation more straightforward, two -OR groups are set as two -OH groups. Therefore, O2 beads are two -OH groups in MC, O3 beads are two -OH groups in Alg, and O4 beads are two -OH groups in Cts. Although the three beads are the same, to distinguish them in different wall materials, different numbers are set. The interaction parameters of the three beads with other beads are the same.

All simulations start from the random distribution of each component. The simulation is carried out in a cube box with periodic boundary conditions, and its side length is 200 × 200 × 200 Å^3^. After calculations, the average volume of the beads is 111.03 Å^3^, the mass is 54 amu, and the radius is 2.98 Å. In the simulation system, the number density $\rho r_{c}^{3}$ of the beads is set to 3, and there are three beads in each grid. That is, the grid size is 333.09 Å^3^, the side length is 6.93 Å, and the cut-off radius *r_c_* between the beads is calculated to be 6.93 Å. The polymerization degree is set to 20. According to the pre-simulation experimental results, there is almost no difference between the sections obtained by 250,000 steps and those obtained by 200,000 steps, indicating that the system has reached the microphase separation equilibrium when the simulation time is 200,000 steps. In order to ensure the accuracy of the results, the simulation time of all systems is 250,000 steps. The time integration step is 0.05 ns. All simulations are carried out in the Mesosite module of Materials Studio 7.0 software [40]. To clearly observe the shape of the beads in the box, all the results hide the water beads.

### 2.3. Simulation System and Scheme

The simulated system consists of citral, Cts, Alg, MC, and H_2_O. First, Cts–Citral single-wall microcapsules are prepared by adding different volume fractions of Cts. Then, in the case of fixed Cts, different volume fractions of Alg or MC are added to replace part of the Cts to prepare double-wall microcapsules. By observing the formation process of the microcapsule and analyzing the simulation results, the influence of the content of each component on the formation of Cts microcapsules is found, which provides guidance for the preparation of Cts microcapsules with better performance.

It has been reported [41,42] that under the experimental conditions of 1.0 g/30 mL H_2_O, 6.0 g/180 mL H_2_O, and 1.6 g/50 mL H_2_O for Cts, Alg, and MC, the content of citral is the largest, and the encapsulation performance and stability of microcapsules are better. The following simulation scheme is designed. In each simulation system, the volume of water is 260 mL, and the volume of citral is 1.5 mL. The amount of the remaining components and the volume fraction of all components are shown in Table 2, Table 3, Table 4 and Table 5.

## 3. Results and Discussion

### 3.1. Self-Assembly Process of Microcapsules

**Figure 2:**
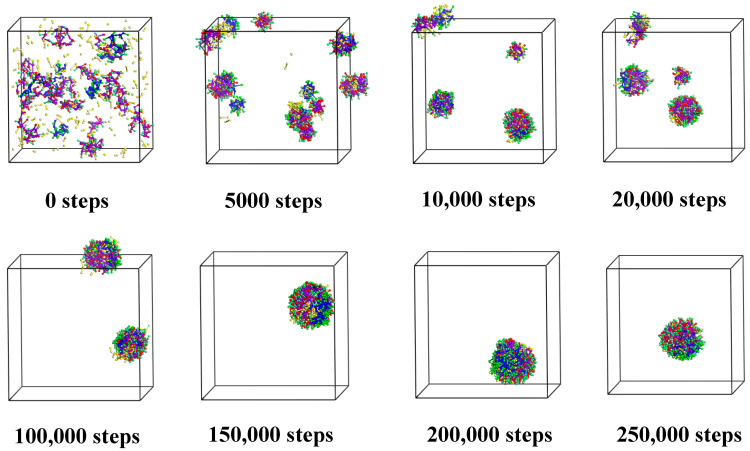
Formation of microcapsules.

**Figure 3:**
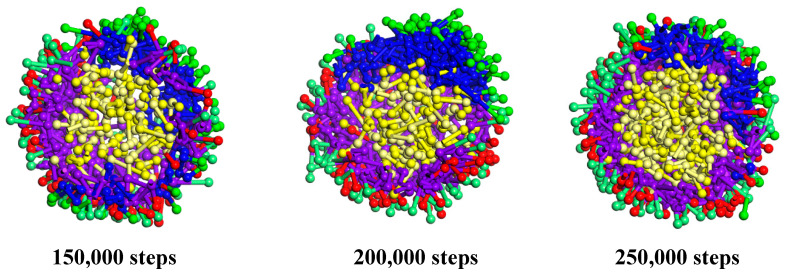
Cross section views of 150,000, 200,000, and 250,000 steps.

**Figure 4:**
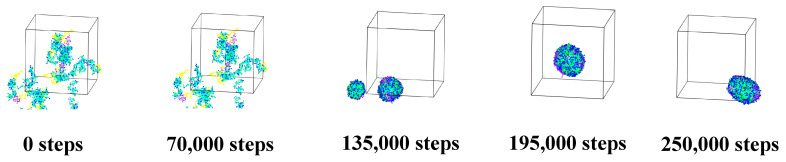
Self-assembly of microcapsules made of Alg and Cts double-wall materials. (Periodic boundary conditions have been removed for clarity. The images represent the microcapsule structure at specific simulation time steps).

To ensure the balance of the system, the DPD simulation steps are set to 250,000 steps. Every 5000 steps is a frame, so the experiment has a total of 51 frames. The formation process of microcapsules can be seen from the simulation results, as shown in Figure 2, Figure 3 and Figure 4. To facilitate observation, the water beads are hidden here. The stability of the microcapsules is supported by their consistent spherical shape and uniform size distribution, as observed in previous studies [38,43]. The radius of gyration and shape parameters were found to be consistent with those reported in similar systems.

#### 3.1.1. Cts–Alg Double-Wall Microcapsule

From the microcapsule formation process shown in Figure 2, it can be seen that at the beginning, the molecules are randomly dispersed in the box. At 5000 steps, some of the molecules combine with the same kind of molecule and curl up with each other. Most of the molecules are close to each other, forming aggregates of different sizes, and some of the aggregates contain citral molecules. At 20,000 steps, the former small agglomerations further merge and become larger and gradually become spherical. By 150,000 steps, all the components have gathered together to form a spherical aggregate, namely, a microcapsule. However, the aggregation degree of each component cannot be clearly judged from the appearance, so the aggregates at 150,000, 200,000, and 250,000 steps are cut open to observe the cross-section, as shown in Figure 3.

To quantitatively evaluate the structural integrity and defects of the microcapsules, we analyzed the radial distribution function (RDF) [44] and concentration profiles at different simulation steps. While radial density profiles provide valuable information about the distribution of species from the center-of-mass, the RDF was chosen to highlight the relative distances between different components, which is crucial for understanding the encapsulation mechanism. The results show that at 150,000 steps, the structure of the microcapsules is not yet fully stable, with significant fluctuations in the RDF curves, indicating that intermolecular interactions have not reached equilibrium. However, at 200,000 and 250,000 steps, the RDF curves stabilize, indicating that the system has reached microphase separation equilibrium.

#### 3.1.2. Alg–Cts Double-Wall Material Microcapsule

As shown in Figure 4, the simulation results show that the Alg, Cts, and citral in the initial state (0 step) disperse irregularly in the water, and the polymer exhibits a random stretching state. With the development of the simulation, the citral in the aqueous phase formed small aggregates under the influence of hydrophobicity and gradually approached the polymer. With increasing simulation time, the aggregates continue to collide further and diffuse into the polymer at the same time. In 195,000 steps, single and relatively uniform spherical nanoparticles are formed. It can be clearly and directly seen that Alg and Cts can be used as composite wall materials of citral microcapsules, which provides a research basis for practical applications.

#### 3.1.3. Alg–MC Double-Wall Microcapsule

**Figure 5:**
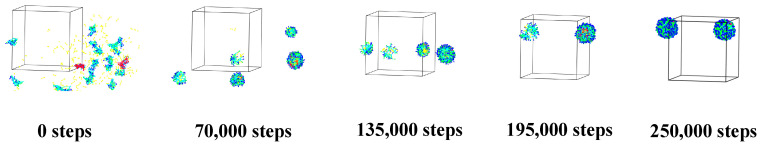
Self-assembly of microcapsules made of Alg and MC double-wall material. (Periodic boundary conditions have been removed for clarity. The images represent the microcapsule structure at specific simulation time steps).

The number of simulation steps is 250,000, and there are 51 frames per 5000 steps. The following is taken from frames 1, 14, 27, 39, and 51 of the track file (xtd file) obtained from the fourth group of Alg–MC oil, and the results are shown in Figure 5. To observe the experimental results, the water beads are hidden. The simulation results show that Alg, MC, and citral in the initial state (0 step) disperse irregularly in water, and that the polymer exhibits a random stretching state. With the progress of the simulation, the polymer in the aqueous phase forms small aggregates under the influence of hydrophobicity and gradually approaches other polymers. As the simulation time continues to increase, the aggregates continue to collide further and diffuse into other polymers at the same time. By frame 39, complete particles have been formed. At frame 39, there is a multiparticle in a small box. When increasing the size of the box, it can be observed that 1.5 boxes can form two complete microspheres, as shown in frame 51. It is clear that Alg and MC can be used as composite wall materials of citral microcapsules, which provides a research basis for the practical application of composite wall materials.

### 3.2. Structure Analysis of Microcapsules

The ideal microcapsule structure should have good stability and intelligent aggregation to improve its sealing, which is very important to obtain a high small-molecule encapsulation ability. Therefore, first, the morphology and cross-section of microcapsules are analyzed to evaluate the distribution and aggregation of beads in microcapsules. Then, the encapsulation and bead distribution of microcapsules with different wall materials are studied more specifically and deeply from the RDF, and the internal structure and morphology of microcapsules are analyzed by comparing RDF curves.

#### 3.2.1. Single-Wall Material

##### Cts–Citral Single-Wall Microcapsule

**Figure 6:**
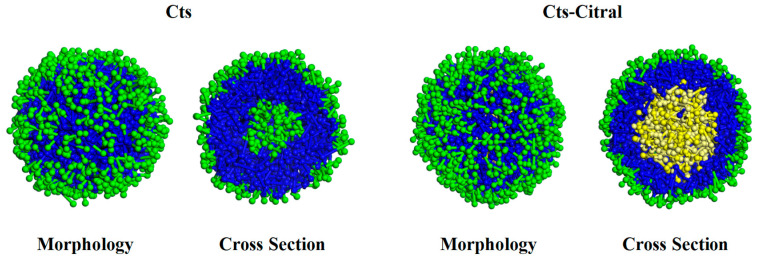
Morphology and cross-section of Cts microspheres and microcapsules.

As shown in Figure 6, Cts microspheres are divided into three layers, namely O4 beads, N1 beads, and O4 beads, from the outside to the inside. The reason for the formation of such a structure is that O4 is a hydrophilic hydroxyl group, so it is distributed in the outermost layer adjacent to water molecules. The main chain of Cts is a hydrophobic carbon chain, which should be distributed far from water beads, and all of them should be in the interior. However, because Cts is a high-molecular-weight polymer, the space volume of the main chain is relatively large, so it cannot be fully squeezed in the middle of the microsphere. Therefore, the main chain is distributed in the middle layer. At the same time, a small part of the branch chain, that is, O4 beads, extends into the inner core, and the innermost layer is a small part of the branch chain, that is, O4 beads extend into it. Therefore, a three-layer structure with a hydrophilic core and shell layers and a hydrophobic medium layer is formed.

When the core material citral was added, a Cts–Citral microcapsule was formed. Morphologically, the Cts–Citral microcapsule is also a three-layer structure. From the outside to the inside of the microcapsule, O4 beads of Cts, N1 beads of Cts, and C1 and C2 beads of citral are in order. Citral has a great repulsive force with water molecules, so it is distributed in the innermost layer. The N1 bead in the main chain of Cts is a six-membered ring structure with a lipophilic carbon chain, so the N1 bead is adjacent to citral and is in the middle layer of the microcapsule. The O4 bead of the Cts branch chain is a hydrophilic group, and there is also a large repulsive force between the O4 bead and C1 and C2 beads, so O4 beads will not be adjacent to the C1 and C2 layers, but all extend to the outside. From Figure 4, when citral is added, the coating layer of the microcapsule becomes thinner. The reason is that the volume of the inner layer becomes larger, which leads to more compact binding between the main chains of Cts.

**Figure 7:**
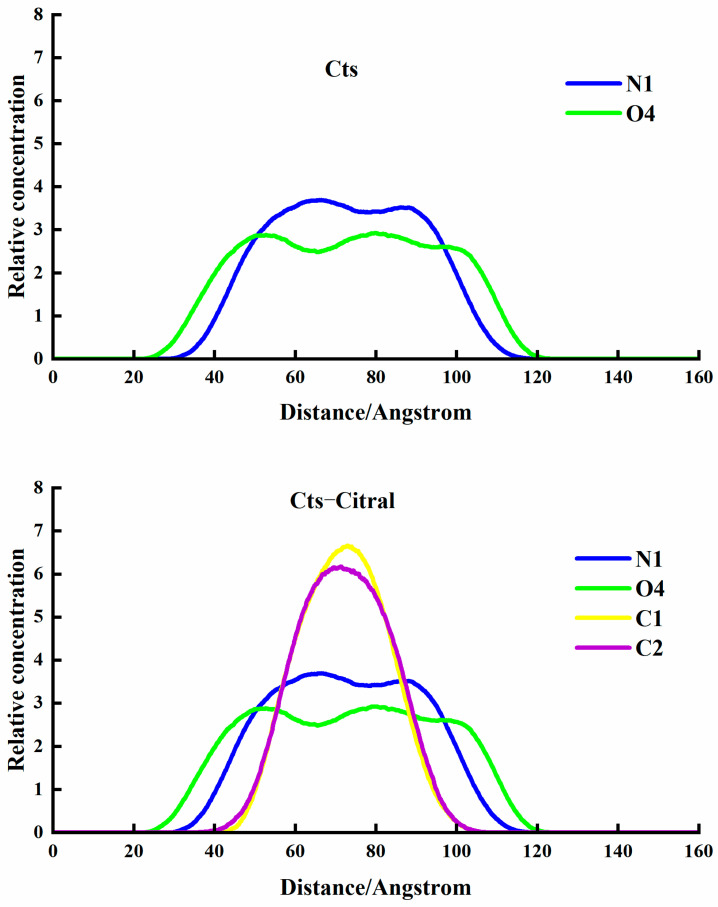
Concentration distribution curve of Cts microcapsules and microspheres.

It can also be seen from the concentration distribution curve in Figure 7 (The concentration profiles were calculated by radially averaging the bead density from the center-of-mass of the microcapsule, with a bin width of 1 Å) that when there is only the wall material Cts, the concentration distribution curve of O4 beads has a peak at 80 Å in the middle, indicating that the concentration of O4 beads is the highest in the middle, which confirms that the O4 beads are distributed in the innermost layer. Because of the symmetry of the microsphere, the curves of N1 and O4 are approximately symmetrical. The middle part of the O4 bead peak is clearly depressed, which indicates that most of the innermost O4 beads are extruded due to the entry of citral; therefore, the concentration of O4 beads is greatly reduced. In addition, the peaks of the N1 and O4 beads are wider than those of the C1 and C2 beads, which further explains the microcapsule structure.

(a) Effect of Cts content on the appearance and cross-section of Cts–Citral microcapsules

**Figure 8:**
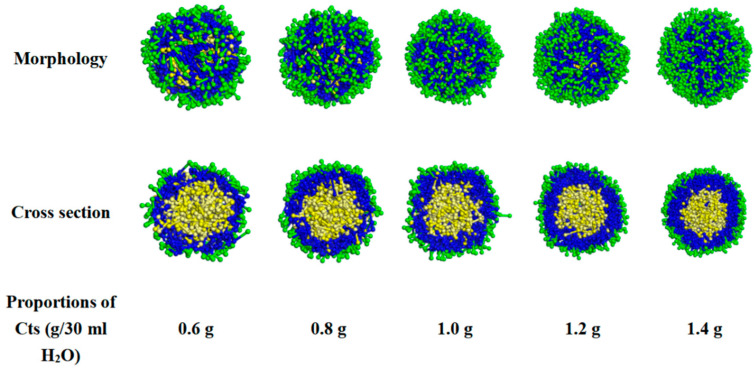
Appearance and cross-section of DPD simulations with different proportions of Cts to H_2_O.

By changing the content of Cts, the cross-section and RDF of the microcapsule are compared to analyze the effect of Cts content on the properties of the microspheres. As shown in Figure 6, the water volume is set as 260 mL, the citral volume is 1.5 mL, and the concentration of Cts is set as 0.6 g/30 mL H_2_O, 0.8 g/30 mL H_2_O, 1.0 g/30 mL H_2_O, 1.2 g/30 mL H_2_O, and 1.4 g/30 mL H_2_O. The simulation results are shown in Figure 8. The volume fraction is calculated, and the corresponding simulations are carried out.

Figure 8 shows that when the Cts content is 0.6 g/30 mL H_2_O, the microspheres have obvious defects in appearance. The microsphere is not wrapped well enough to even see the citral molecules inside. The cross-section shows that some citral molecules are embedded in the main chain of Cts. Therefore, the microcapsules under this condition are not complete, the thickness of the cladding is small, and the arrangement of O4 beads is not neat enough. These disadvantages will seriously reduce the encapsulation performance of Cts microcapsules, which makes disintegration easy during storage.

When the content of Cts is increased to 0.8 g/30 mL H_2_O and 1.0 g/30 mL H_2_O, the cracks of the shell tended to close, and few citral molecules are exposed. The cross-section shows that the citral molecule shrinks inward, and that the citral molecule embedded in the main chain layer of the Cts molecule gradually detaches. However, from the appearance and cross-sectional point of view, there are still some defects in the shape of the microspheres, which is not a more regular sphere.

When the content of Cts is increased to 1.2 g/30 mL H_2_O, few citral molecules are embedded in the main chain of Cts, and the encapsulation is further improved. The order of O4 beads is greatly improved, and the thickness of the whole microcapsule is more uniform than before. When Cts reaches the maximum content of 1.4 g/30 mL H_2_O in the simulation experiment, the citral molecules in Cts are almost invisible. The whole sphere is round, and there is no citral molecule embedded in the main chain of Cts. The Cts microcapsule layer is thicker than the core material. The arrangement of O4 beads is also very neat, forming a three-layer structure of core material–N1–O4. Clearly, the Cts microcapsule with this concentration has the best encapsulation performance.

(b) Analysis of RDF

**Figure 9:**
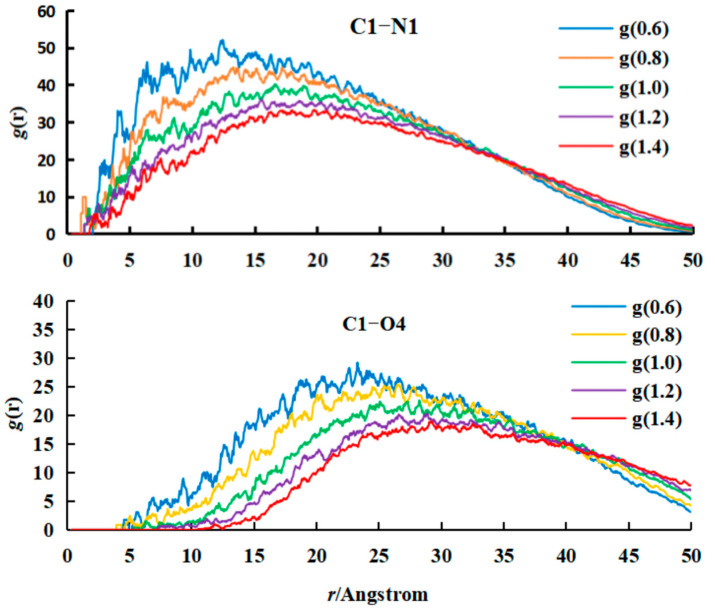
RDF of N1 and O4 beads with different proportions of Cts.

The RDF refers to the distribution probability of other particles relative to a given particle in space, so the RDF can reflect the distance relationship between particles. In this study, C1 beads in citral molecules are selected as reference beads, and then the RDFs of the N1 and O4 beads in Cts relative to C1 beads are studied and compared, as shown in Figure 9. From the RDF curves of C1–N1 and C1–O4, the peak of the C1–N1 curve is higher and closer to the left end than that of C1–O4, indicating that the distance between the N1 bead and C1 bead is closer. That is, the affinity between the C1 and N1 beads is better than that between the C1 and O4 beads, and the interaction force is smaller. This conclusion is consistent with the size of the interaction parameter a_ij_ calculated above, which indicates that the RDF curve can reflect the size of the interaction parameter of each component from the side.

From the RDF curve of C1–N1, it can be seen that with increasing Cts content, the peak of the C1–N1 RDF curve gradually decreased and shifted to the right. The change trend of the RDF curve showed that the higher the content of Cts is, the greater the N1 beads spread to the outer layer; that is, the distance between the C1 and N1 beads became larger. The reason for this is that with increasing Cts molecules, the affinity between the N1 and C1 beads decreases, and the repulsive force increases. However, the space volume of the N1 bead is so large that it cannot diffuse to the inside, only to the outside. The RDF curve of C1–O4 is very similar to that of C1–N1. With increasing Cts content, the peak of the curve gradually decreased and shifted to the right. The reason is that the N1 beads diffused to the outer layer under the gradually strengthened repulsive force, so the O4 bead is forced to move to the outer layer as a whole. This analysis can also explain why the C1 beads gradually separate from the N1 beads with increasing Cts content in the cross-section. Because the affinity between the C1 and N1 beads decreased and the repulsive force increased, the C1 beads could not be embedded into the N1 beads.

By comparing the above groups of simulation experiments, it can be found that when the content of citral is fixed, the higher the content of Cts is, and the better the encapsulation performance of citral. However, considering the cost and other factors, the content of Cts should be controlled within a certain range, so it is not necessary to increase it too much, as long as it can be completely wrapped with citral.

In this study, the RDFs were averaged over the last 50,000 simulation steps (500 frames sampled every 100 steps) to ensure statistical reliability.

##### Alg–Citral Single-Wall Microcapsule

**Figure 10:**
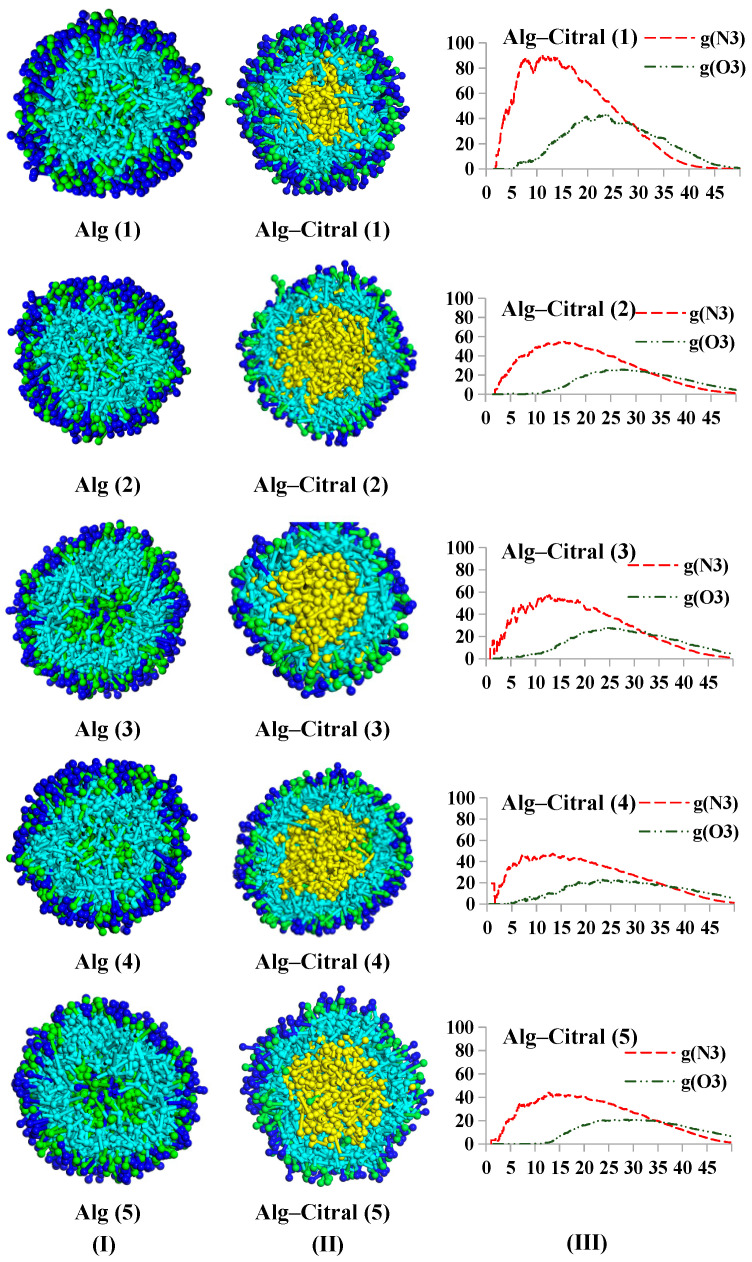
Alg single-wall microcapsule ((**I**) citral free; (**II**) containing citral; and (**III**) RDF curve).

Figure 10 shows that under neutral simulation conditions, Alg can form a spherical core–shell structure and tightly embed the citral in it. The hydrophilic O3 beads (dark blue) are located in the outermost layer of the microcapsule, forming a hydrophilic protective shell, and N3 is mainly in the hydrophobic inner layer and tightly envelops the citral.

Compared with the profile of microcapsules, the RDF curve can more directly reflect the distribution characteristics of citral. For the same content of citral, the citral is preferentially distributed in the inner layer (hydrophobic layer) of the capsule under the condition of a lower content of wall material. Because the affinity of N3 hydrophobic beads to citral (C1 beads) is greater than that of O3, the citral is more distributed in the inner layer. With an increase in wall material content until saturation, the extra N3 beads diffuse to the outer layer. Therefore, it can be seen that with increasing wall material content, the peak of the N3 bead curve is closer to that of the O3 bead curve.

#### 3.2.2. Double-Wall Material Microcapsule

##### Cts–Alg–Citral and Cts–MC–Citral Microcapsules

The effects of Alg and MC on Cts–Citral microcapsules are also simulated. DPD simulation with a Cts content of 1.0 g/30 mL H_2_O is selected for comparison, and the total amount of wall materials in the three groups is equal.

(a) Appearance and cross-section of microcapsules

**Figure 11:**
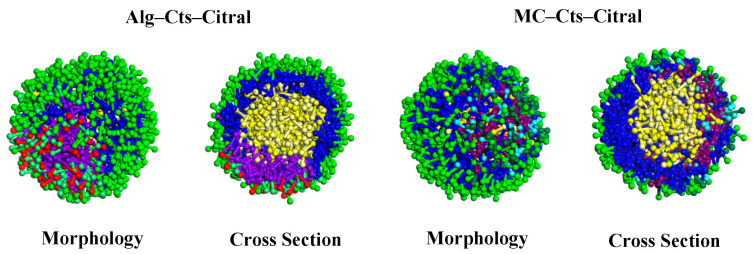
Morphology and cross-section of Alg–Cts–Citral and MC–Cts–Citral microcapsules.

Figure 11 shows that for the microcapsules doped with Alg or MC, both Alg and MC have obvious boundaries with Cts molecules and are not mixed with Cts molecules. The formation of this phenomenon can be explained by the interaction between the molecules of each component. Clearly, the interaction between Cts molecules is weaker than that between Cts and Alg or MC, and the corresponding repulsive forces are stronger.

Compared with the pure Cts microcapsule, the microcapsule made of Cts doped with Alg has no obvious difference in appearance and shape, and a small amount of citral molecules are embedded in the wall layer. However, when Cts doped with MC is used as the wall material, the microcapsule is quite different. First, citral molecules are more exposed in the microspheres formed by MC. Second, from a cross-sectional view, the microspheres formed by MC are obviously thinner, and the citral molecules are embedded more, with some even embedded in the most peripheral position of the microcapsule. Moreover, a thin Cts microsphere layer covered the outside of the MC microcapsule layer. This phenomenon indicates that the encapsulation performance of MC to citral is slightly worse than that of Cts, and it cannot be well coated with citral.

(b) Analysis of the RDF

**Figure 12:**
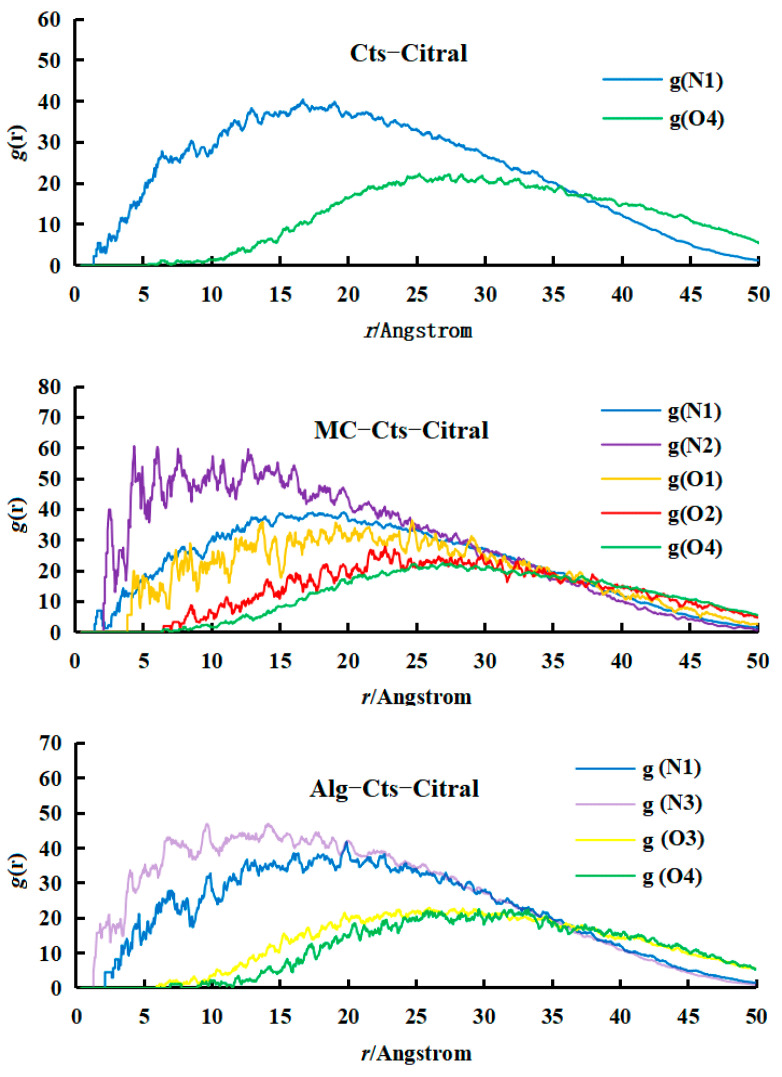
RDF curves of Cts–Citral, Alg–Cts–Citral, and MC–Cts–Citral microcapsules.

The results are shown in Figure 12, and the RDF curves in the figure are all based on C1 beads. Curve g(N1) represents the RDF of C1–N1 and curve g(O1) represents the RDF of C1–O1, and so on.

By comparing the three figures, the RDFs of the C1–N1 beads and C1–O4 beads are basically the same, and there is no obvious fluctuation. That is, adding Alg or MC to replace part of Cts has little effect on the molecular distribution of Cts in the microcapsule wall.

On the other hand, the peak of the C1–N2 curve is much higher than those of the C1–N1 and C1–N3 curves, which indicates that the affinity of N2 beads in the main chain of MC with C1 beads of citral is stronger than that with N1 beads of Cts or N3 beads of Alg. Therefore, compared with Cts and Alg, there are more citral molecules embedded in the molecular layer of MC.

In addition, the peak of the C1–O1 curve is much higher than those of C1–O2, C1–O3, and C1–O4, and the whole C1–O1 curve is closer to the left than the other three curves. The curve characteristics showed that the affinity between the O1 and C1 beads is stronger than that between the O2, O3, and O4 beads.

(c) Analysis of the concentration distribution curve of citral

**Figure 13:**
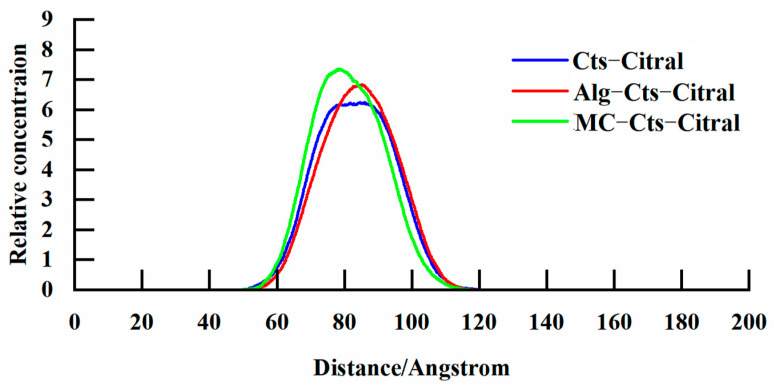
Concentration distribution curve of citral molecules.

As shown in Figure 13, in the system doped with Alg, the peak of the concentration distribution curve of citral is higher than that of the system without Alg, but the upper part of the peak is narrower than that of the pure Cts wall material. The area enclosed by the concentration distribution curve of the two is almost the same, which indicates that the content of the coated citral is very close. However, in the system doped with MC, the peak of the concentration distribution curve of citral is higher than those of the other two, but the width is almost the same, so the area surrounded by the concentration distribution curve is larger, which indicates that the Cts wall material doped with MC has more citral content. However, the width of the bottom of the three curves is almost the same, which indicates that the volume of microcapsules formed by the three groups of experiments is similar, and that there is little difference. Under the condition of almost no change in volume, the content of citral in the system doped with MC increases, which may be because the addition of MC weakens the interaction between the molecules and makes the molecules more closely arranged, so the volume of the system is almost unchanged.

It can be seen from the above discussion that the doping of Alg has no obvious effect on the preparation of Cts–Citral microcapsules because the properties of the microcapsules have not changed much. It is concluded that Alg can replace Cts in the preparation of Cts–Citral microcapsules. On the other hand, the doping of MC has a significant effect on the preparation of Cts–Citral. Because MC is amphiphilic and has a strong affinity for citral, more citral can be encapsulated in it. However, the encapsulation performance of MC is not very good, which easily leads to the exposure of citral molecules. Therefore, there are advantages and disadvantages for the strategy of doping MC.

###### Alg–Cts–Citral Double-Wall Material Microcapsule of Alg and Cts

**Figure 14:**
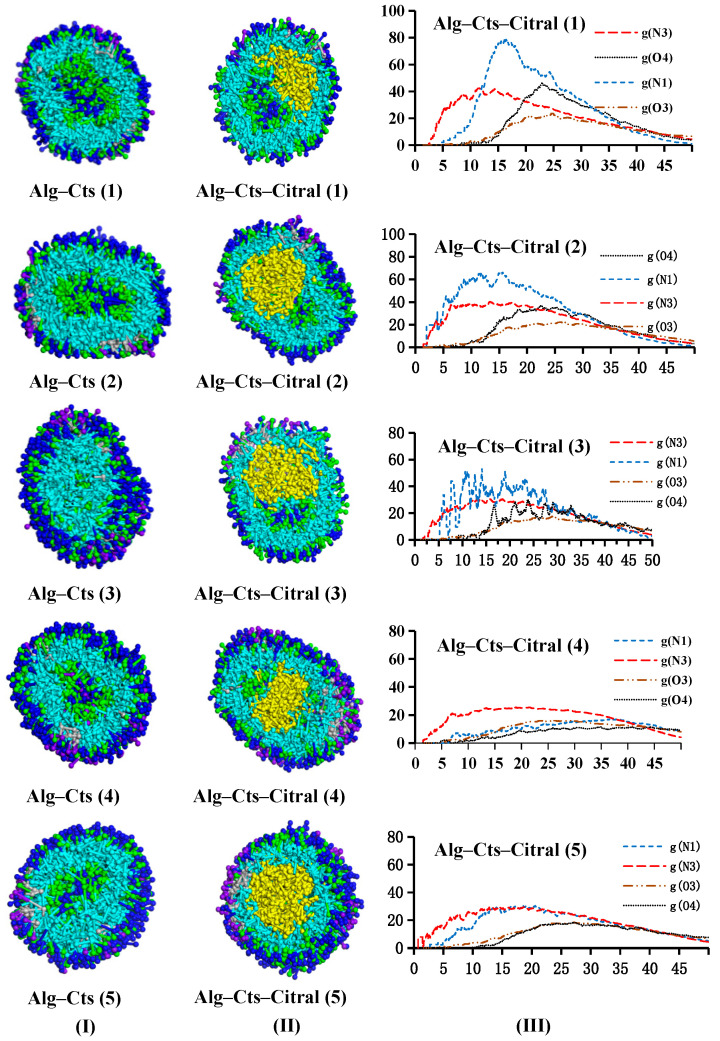
Alg and Cts double-wall material microcapsules ((**I**) citral free; (**II**) containing citral; and (**III**) RDF curve).

Figure 14 shows that under neutral simulation conditions, Alg and Cts can form a spherical core–shell structure, which can tightly embed the citral in it. The N1 beads and O4 beads of Cts are mainly distributed in the outer and middle layers. The bead distribution of Alg is similar to that of single-wall Alg. Hydrophilic O3 beads (dark blue) are distributed in the outermost layer of the microcapsule, forming a hydrophilic protective layer. S beads are distributed in the middle and outer layers of the microcapsules, while N3 beads are next to the citral.

The RDF curve can reflect the distribution characteristics of citral in wall materials more directly. Under the condition of keeping the content of citral and Alg unchanged, when the content of Cts is low, N1 and N3 beads are preferentially distributed in the inner layer close to citral. The reason is that the affinity of citral to hydrophobic beads (N1 and N3 beads) is greater than that to hydrophilic beads (O3 and O4 beads). With increasing Cts content in the wall material, the inner layer gradually becomes saturated. At the same time, the space resistance of hydrophobic beads to diffuse to the outer layer of the capsule may be less than the attraction of affinity, so hydrophobic beads gradually distribute to the outer layer, which leads to the coincidence of the peaks of hydrophobic beads and hydrophilic beads. As shown in Figure 14III, the peak coincidence degree of all curves in group (4) is the highest, indicating that Cts and Alg are closely bound. The reason for this may be that there is a strong electrostatic interaction between Cts and Alg, so the compactness and stability of the composite wall material are better. Therefore, the best experimental results are obtained for the feeding ratio of group (4). It is concluded that when the contents of citral, Alg, and Cts are 0.55%, 3.25%, and 0.25%, that is, after dehydration the microcapsules have better performance when the contents of citral, Alg, and Cts are 13.6%, 80.2%, and 6.2%, respectively. 

##### Alg–MC–Citral Double-Wall Material Microcapsule

**Figure 15:**
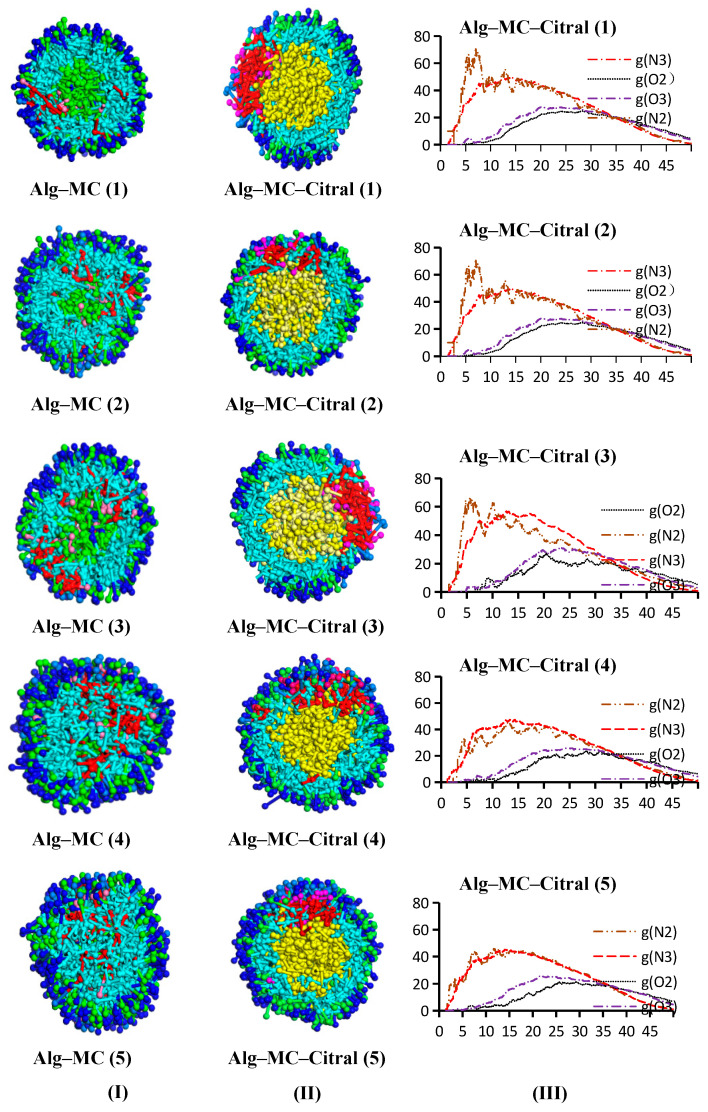
Alg and MC double-wall material microcapsules ((**I**) citral free; (**II**) containing citral; and (**III**) RDF curve).

Figure 15 shows that in aqueous solution, Alg and MC can also form a spherical core–shell structure, which can tightly embed the citral. The N2 beads and O2 beads of MC are distributed in the outer and middle layers. The bead distribution of Alg in aqueous solution without citral is similar to that of single wall Alg. Hydrophilic O3 beads (dark blue) are used as the outermost layer of the microcapsule to form a hydrophilic protective layer and S beads are distributed in the middle and outer layers of the microcapsules. In the model containing citral, the N3 bead layer is close to the citral.

Compared with the profile of microcapsules, the RDF curve can more directly reflect the distribution characteristics of citral and wall material. Under the condition of keeping the content of citral and Alg unchanged, the content of MC is gradually increased to investigate the change in bead distribution. When the content of MC is low, N2 and N3 beads are preferentially distributed in the inner layer near citral, because citral has more affinity for hydrophobic N2 and N3 beads than for hydrophilic O2 and O3 beads. When the content of MC increases, the total content of the wall material also increases, and the hydrophobic layer tends to be saturated so that the probability of hydrophobic beads distributed in the hydrophobic layer and hydrophilic layer gradually tends to be equal. Clearly, the peak value of hydrophobic beads is close to that of hydrophilic beads, but there is still a certain gap. This may be due to the amphiphilic property of MC, which has an obvious solubilization effect on citral. The hydrophobic beads of Alg (N3 beads) and the hydrophobic group of MC (N2 beads) are more likely to be distributed in the inner layer, while the hydrophilic beads of Alg (O3 beads) and the hydrophilic group of MC (O2 beads) are more likely to be distributed in the outer layer. In group (5), the peak value of the hydrophobic bead curve is higher, but the difference in the peak value of the hydrophilic layer is larger, which indicates that the hydrophilic effect between the two wall materials is not very strong. In contrast, the two peaks of group (4) are very close, which indicates that the interaction between the two wall materials is the strongest, and that the combination degree and encapsulation effect are the best. Therefore, group (4) exhibits the best result. Therefore, when the contents of citral, Alg, and MC are 0.56%, 1.65%, and 0.29%, respectively, that is, after dehydration the microcapsules demonstrate better performance when the contents of citral, Alg, and MC are 22.4%, 66.0%, and 11.6%, respectively.

##### Diffusion Coefficient of Citral in the Simulation Process

**Figure 16:**
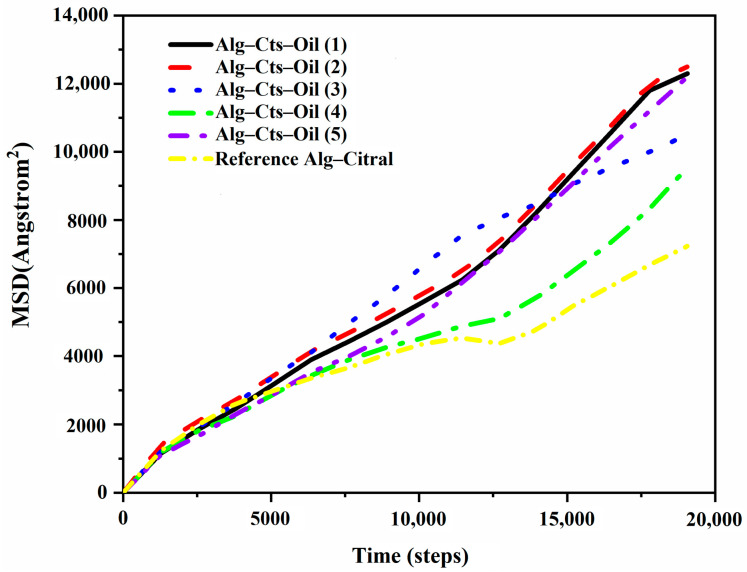
Mean square displacement function trend diagram of Alg and Cts double-wall materials.

MSD is the distance the beads move from their original position to the second moment of their distribution in a defined time span, defined as $MSD=\frac{1}{N}\sum_{i=1}^{N}{\left|r_{i}(t)-r_{i}(0)\right|}^{2}$, which is related to the diffusion coefficient ($F_{D}=\frac{1}{6N}\underset{t\to \infty }{\lim}\frac{d}{dt}\sum_{i=1}^{N}{\left|r_{i}(t)-r_{i}(0)\right|}^{2}$), where *r_i_* denotes the position vector of *i*th bead and *N* is the number of statistical beads [45]. The MSD curves are also computed in the Mesocite module of Materials Studio 7.0 software. The slope of the mean square displacement diagram can represent the diffusion coefficient. A large slope indicates a fast diffusion speed [38,43]. Figure 16 is a trend line diagram of the mean square displacement. The diffusion coefficient can be directly seen from the inclination angle of the slash. Clearly, the diffusion coefficient of the fourth group is small and close to that of the Alg single-wall material system. The slopes of the other groups hardly changed, which may be because the fourth group of Cts cations reacted completely with Alg anions. However, when the amount of Cts is too small or too large, the reaction between the Cts cation and Alg anion is not complete. Therefore, the system has excess anions or cations, resulting in electrification of the simulated system, and the formed microspheres are also unstable. Therefore, the diffusion coefficient of other groups becomes higher, and the diffusion speed of citral becomes faster.

**Figure 17:**
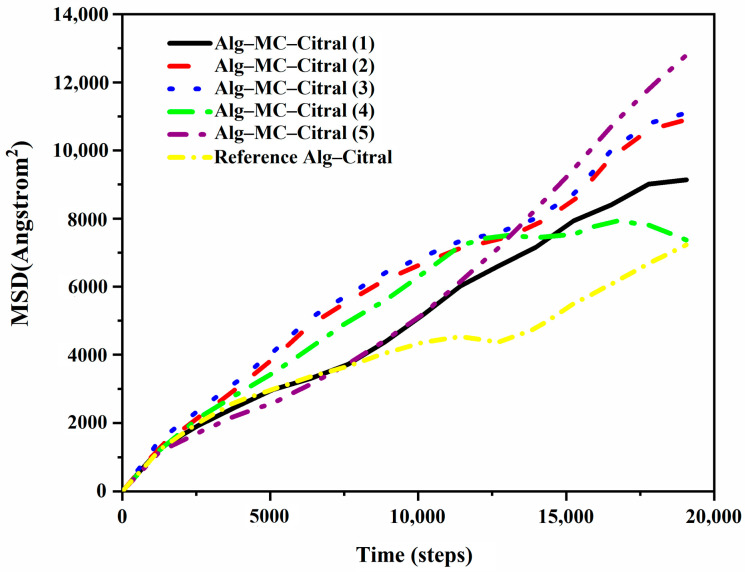
Mean square displacement function trend diagram of Alg and MC double-wall materials.

As shown in Figure 17, in the simulation results of the Alg and MC composite wall material, the diffusion coefficient of the fourth group is close to that of the Alg single-wall material system. However, the diffusion coefficients of the other groups are large and almost the same. This may be because MC is amphiphilic and has an obvious solubilization effect on citral [46]. When the content of MC is high, the diffusion coefficient becomes higher, and the diffusion speed of citral becomes faster. When the content of MC is relatively small, according to the RDF in this paper, the wall material may be loose, resulting in a higher diffusion coefficient. The MSD curves exhibit different regimes, reflecting the initial rapid diffusion of components followed by a slower, more stable phase. The decreasing trend observed in Alg–MC–Citral (4) is likely due to the strong electrostatic interactions between the components, which temporarily restrict their motion before reaching equilibrium.

## 4. Conclusions

In this study, the DPD method is used to simulate the self-assembly process, appearance, mesoscopic structure, and wrapping properties of microcapsules formed with citral as the core material and Cts and Alg as single-wall materials, and with citral as the core material and Cts–Alg, Cts–MC, Alg–Cts, and Alg–MC as double-wall materials. The effects of Cts content and wall material composition on the structure, morphology, encapsulation performance, and stability of microcapsules are compared and explored. In addition, the microcapsules are deeply analyzed by using the mesoscopic structure, RDF, and diffusion coefficient.

The conclusions are as follows: (1) The results show that the higher the Cts content, the better the coating performance of the wall material on citral. However, considering the cost of the actual production process and other factors, the dosage of Cts should not be too high, as long as it can completely wrap citral. (2) When Alg–Cts double-wall microcapsules are prepared with water as the solvent, the microcapsules have better performance when the contents of citral, Alg, and Cts are 0.55%, 3.25%, and 0.25%, respectively. (3) When Alg–MC double wall microcapsules are prepared with water as the solvent, the microcapsules demonstrate better performance when the contents of citral, Alg, and MC are 0.56%, 1.65%, and 0.29%, respectively.

This study provides a new idea and method for the preparation of citral microcapsules and is of great significance for the design and development of new composite wall microcapsules.

## Figures and Tables

**Table 1:** Interaction parameters among beads.

*a_ij_*	N1	N2	O1	O2	C1	C2	S	W
N1	25							
N2	25.92	25						
O1	27.47	30.81	25					
O2	44.23	51.46	31.06	25				
C1	29.05	26.08	36.27	61.69	25			
C2	25.49	25.01	29.65	46.44	25.16	25		
S	31.77	36.46	25.88	27	43.43	34.17	25	
W	95.99	106.18	57.89	35.74	121.98	87.94	44.73	25

Note: All parameters in Table 1 are expressed in DPD reduced units, where *r_c_* = 1, *k_B_T* = 1, and *ρ* = 3.

**Table 2:** Amounts and volume fractions of each component of Cts single-wall microcapsules.

	Number	H_2_O (mL)	*m* (Cts) (g)	*φ* (H_2_O)	*φ* (Cts)	Citral (mL)	*φ* (Citral)
Citral free	Cts (1)	260.00	5.2	98.87	1.13		
	Cts (2)	260.00	6.93	98.50	1.50		
	Cts (3)	260.00	8.67	98.13	1.87		
	Cts (4)	260.00	10.4	97.77	2.23		
	Cts (5)	260.00	12.13	97.40	2.60		
Containing Citral	Cts–Citral (1)	260.00	5.2	98.31	1.12	1.5	0.57
	Cts–Citral (2)	260.00	6.93	97.94	1.49	1.5	0.57
	Cts–Citral (3)	260.00	8.67	97.58	1.86	1.5	0.56
	Cts–Citral (4)	260.00	10.4	97.22	2.22	1.5	0.56
	Cts–Citral (5)	260.00	12.13	96.86	2.58	1.5	0.56

**Table 3:** Amounts and volume fractions of each component of Alg single-wall microcapsules.

	Number	H_2_O (mL)	Alg (g)	*φ* (H_2_O)	*φ* (Alg)	Citral (mL)	*φ* (Citral)
Citral free	Alg (1)	260.00	7.22	98.28	1.72		
	Alg (2)	260.00	7.94	98.12	1.88		
	Alg (3)	260.00	8.67	97.95	2.05		
	Alg (4)	260.00	9.39	97.78	2.22		
	Alg (5)	260.00	10.11	97.61	2.39		
Containing Citral	Alg–Citral (1)	260.00	7.22	97.73	1.71	1.5	0.56
	Alg–Citral (2)	260.00	7.94	97.56	1.87	1.5	0.56
	Alg–Citral (3)	260.00	8.67	97.40	2.04	1.5	0.56
	Alg–Citral (4)	260.00	9.39	97.23	2.21	1.5	0.56
	Alg–Citral (5)	260.00	10.11	97.07	2.37	1.5	0.56

**Table 4:** Amounts and volume fractions of each component of the Alg–Cts double-wall microcapsule (the amount of Alg is fixed at 14.00 g).

	Number	H_2_O (mL)	Alg (g)	*φ* (H_2_O)	Cts (g)	*φ* (Cts)	*φ* (Alg)	Citral (mL)	*φ* (Citral)
Citral free	Alg–Cts (1)	260.00	14.00	96.60	0.60	0.13	3.27		
	Alg–Cts (2)	260.00	14.00	96.56	0.80	0.17	3.27		
	Alg–Cts (3)	260.00	14.00	96.52	1.00	0.21	3.27		
	Alg–Cts (4)	260.00	14.00	96.48	1.20	0.25	3.27		
	Alg–Cts (5)	260.00	14.00	96.44	1.40	0.30	3.27		
Containing Citral	Alg–Cts–Citral (1)	260.00	14.00	96.07	0.60	0.13	3.25	1.50	0.55
	Alg–Cts–Citral (2)	260.00	14.00	96.03	0.80	0.17	3.25	1.50	0.55
	Alg–Cts–Citral (3)	260.00	14.00	95.98	1.00	0.21	3.25	1.50	0.55
	Alg–Cts–Citral (4)	260.00	14.00	95.94	1.20	0.25	3.25	1.50	0.55
	Alg–Cts–Citral (5)	260.00	14.00	95.90	1.40	0.30	3.25	1.50	0.55

**Table 5:** Simulation of Alg–MC double-wall microcapsules (Alg unchanged).

	Number	H_2_O (mL)	Alg (g)	MC (g)	*φ* (H_2_O)	*φ* (MC)	*φ* (Alg)	Citral (mL)	*φ* (Citral)
Citral free	Alg–MC (1)	260.00	7.00	0.40	98.22	0.12	1.66		
	Alg–MC (2)	260.00	7.00	0.60	98.16	0.17	1.66		
	Alg–MC (3)	260.00	7.00	0.80	98.11	0.23	1.66		
	Alg–MC (4)	260.00	7.00	1.00	98.05	0.29	1.66		
	Alg–MC (5)	260.00	7.00	1.20	97.99	0.35	1.66		
Containing Citral	Alg–MC–Citral (1)	260.00	7.00	0.40	97.67	0.12	1.65	1.50	0.56
	Alg–MC–Citral (2)	260.00	7.00	0.60	97.61	0.17	1.65	1.50	0.56
	Alg–MC–Citral (3)	260.00	7.00	0.80	97.55	0.23	1.65	1.50	0.56
	Alg–MC–Citral (4)	260.00	7.00	1.00	97.50	0.29	1.65	1.50	0.56
	Alg–MC–Citral (5)	260.00	7.00	1.20	97.44	0.35	1.65	1.50	0.56

## Data Availability

The original contributions presented in the study are included in the article, and further inquiries can be directed to the corresponding authors.

## References

[1] Ye W., Wang N., Hu K., Zhang L., Liu A., Pan C.J., Gong T., Liu T., Ding H.Y. (2018). Bio-inspired microcapsule for targeted antithrombotic drug delivery. RSC Adv..

[2] Huang D., Sun M., Bu Y.Z., Luo F., Lin C.Y., Lin Z.Y., Weng Z.Q., Yang F., Wu D.C. (2019). Microcapsule-embedded hydrogel patches for ultrasound responsive and enhanced transdermal delivery of diclofenac sodium. J. Mater. Chem. B.

[3] Popov A.L., Popova N., Gould D.J., Shcherbakov A.B., Sukhorukov G.B., Ivanov V.K. (2018). Ceria nanoparticles-decorated microcapsules as a smart drug delivery/protective system: Protection of encapsulated P. pyralis luciferase. ACS Appl. Mater. Interfaces.

[4] Vazquez-Gonzalez M., Willner I. (2018). DNA-responsive SiO_2_ nanoparticles.; metal-organic frameworks.; and microcapsules for controlled drug release. Langmuir.

[5] Huang L.Y., Wu K., Zhang R., Ji H.B. (2019). Fabrication of multicore milli- and microcapsules for controlling hydrophobic drugs release using a facile approach. Ind. Eng. Chem. Res..

[6] Wei L.Y., Lu Z.Q., Ji X., Jiang Y.K., Ma L. (2021). Self-assembly of hollow graphene oxide microcapsules directed by cavitation for loading hydrophobic drugs. ACS Appl. Mater. Interfaces.

[7] Wang Y.F., Cheng Q.Q., Liu J., Tariq Z.Z., Zheng Z., Li G., Kaplan D.L., Wang X. (2020). Tuning microcapsule shell thickness and structure with silk fibroin and nanoparticles for sustained release. ACS Biomater. Sci. Eng..

[8] Guo X.Y., Zhao R., Zhang J., Du Y.J., Yang L.G., Chen L.Y., Pang S., Xu Y., Zhang Z.H., Wu X.M. (2019). A microcapsule oil dispersion for the controlled release of 1-methylcyclopropene in an open environment. RSC Adv..

[9] Suraphan N., Fan L.F., Liu B.X., Wu D.C. (2020). Co-delivery of chlorantraniliprole and avermectin with a polylactide microcapsule formulation. RSC Adv..

[10] Zou A.H., Yang Y., Cheng J.G., Garamus V.M., Li N. (2018). Construction and characterization of a novel sustained-release delivery system for hydrophobic pesticides using biodegradable polydopamine-based microcapsules. J. Agric. Food. Chem..

[11] Yang J.L., Zhou Z.Y., Liang Y., Tang J.Y., Gao Y.H., Niu J.F., Dong H.Q., Tang R., Tang G., Cao Y.S. (2020). Sustainable preparation of microcapsules with desirable stability and bioactivity using phosphonium ionic liquid as a functional additive. ACS Sustain. Chem. Eng..

[12] Jia C.H., Huang S.J., Liu R., You J., Xiong S.B., Zhang B.J., Rong J.H. (2021). Storage stability and in-vitro release behavior of microcapsules incorporating fish oil by spray drying. Colloids Surf. A Physicochem. Eng. Asp..

[13] Fotovvat B., Behzadnasab M., Mirabedini S.M., EivazMohammadloo H. (2022). Anti-corrosion performance and mechanical properties of epoxy coatings containing microcapsules filled with linseed oil and modified ceria nanoparticles. Colloids Surf. A Physicochem. Eng. Asp..

[14] Sun J.Y., Li W., Zhan Y.C., Tian L.M., Tian H.L. (2022). Two preparation processes for anti-corrosion and self-healing epoxy coatings containing the poly (calcium alginate) microcapsules loaded with tung oil. Colloids Surf. A Physicochem. Eng. Asp..

[15] Prabaharan M. (2008). Chitosan derivatives as promising materials for controlled drug delivery. J. Biomater. Appl..

[16] Calvo P., Remunan-Lopez C., Vila-Jato J.L., Alonso M. (1997). Novel hydrophilic chitosan-polyethylene oxide nanoparticles as protein carriers. J. Appl. Polym. Sci..

[17] Zhang L., Wang Y.F., Liu H.S., Yu L., Liu X.X., Chen L., Zhang N.Z. (2013). Developing hydroxypropyl methylcellulose/hydroxypropyl starch blends for use as capsule materials. Carbohyd. Polym..

[18] Zhang L., Lu Y.Q., Yue L.N., Li Q., Xiao L.X., Ding X.L., Guan C.R. (2020). Microstructures, physical and sustained antioxidant properties of hydroxypropyl methylcellulose based microporous photophobic films. Int. J. Biol. Macromol..

[19] Dudai N., Weinstein Y., Krup M., Rabinski T., Ofir R. (2005). Citral is a new inducer of caspase-3 in tumor cell lines. Planta Medica.

[20] Rabbani S.I., Devi K., Shivananda T.N. (2004). Studies on antimutagenic effects of citral in mice. J. Food Agric. Environ..

[21] Shen Y.B., Sun Z.F., Guo X.T. (2015). Citral inhibits lipopolysaccharide-induced acute lung injury by activating PPAR-γ. Eur. J. Pharmacol..

[22] Chueca B., Pagán R., García-Gonzalo D. (2014). Oxygenated monoterpenes citral and carvacrol cause oxidative damage in Escherichia coli without the involvement of tricarboxylic acid cycle and Fenton reaction. Int. J. Food Microbiol..

[23] Gilling D.H., Kitajima M., Torrey J.R., Bright K.R. (2014). Mechanisms of antiviral action of plant antimicrobials against murine norovirus. Appl. Environ. Microb..

[24] Bergonzelli G.E., Donnicola D., Porta N., Corthésy-Theulaz I.E. (2003). Essential oils as components of a diet-based approach to management of helicobacter infection. Antimicrob. Agents Chemother..

[25] Hoogerbrugge P.J., Koelman J.M.V.A. (1992). Simulating microscopic hydrodynamic phenomena with dissipative particle dynamics. EPL.

[26] Espanol P., Warren P. (1995). Statistical mechanics of dissipative particle dynamics. Europhys. Lett..

[27] Abu-Nada E., Pop I., Mahian O. (2019). A dissipative particle dynamics two-component nanofluid heat transfer model: Application to natural convection. Int. J. Heat Mass Transf..

[28] Feng Y.H., Zhang X.P., Zhao Z.Q., Guo X.D. (2020). Dissipative particle dynamics aided design of drug delivery systems: A review. Mol. Pharm..

[29] Okuwaki K., Mochizuki Y., Doi H., Kawada S., Ozawac T., Yasuoka K. (2018). Theoretical analyses on water cluster structures in polymer electrolyte membrane by using dissipative particle dynamics simulations with fragment molecular orbital based effective parameters. RSC Adv..

[30] Zhang J., Xu J.C., Wen L.Y., Zhang F.S., Zhang L.J. (2020). The self-assembly behavior of polymer brushes induced by the orientational ordering of rod backbones: A dissipative particle dynamics study. Phys. Chem. Chem. Phys..

[31] Liang X.P., Wu J.Q., Yang X.G., Tu Z.B., Wang Y. (2018). Investigation of oil-in-water emulsion stability with relevant interfacial characteristics simulated by dissipative particle dynamics. Colloids Surf. A Physicochem. Eng. Asp..

[32] Zhou P., Hou J., Yan Y.G., Wang J.Q., Chen W. (2019). Effect of aggregation and adsorption behavior on the flow resistance of surfactant fluid on smooth and rough surfaces: A many-body dissipative particle dynamics study. Langmuir.

[33] Wang X.Y., Santo K.P., Neimark A.V. (2020). Modeling gas-liquid interfaces by dissipative particle dynamics: Adsorption and surface tension of cetyl trimethyl ammonium bromide at the air-water interface. Langmuir.

[34] Zhang J.W., Chen L., Wang A., Yan Z.C. (2020). Dissipative particle dynamics simulation of ionic liquid-based microemulsion: Quantitative properties and emulsification mechanism. Ind. Eng. Chem. Res..

[35] Choudhary M., Kamil S.M. (2020). Phase diagram study of sodium dodecyl sulfate using dissipative particle dynamics. ACS Omega.

[36] Panoukidou M., Wand C.R., Regno A.D., Anderson R.L., Carbone P. (2019). Constructing the phase diagram of sodium laurylethoxysulfate using dissipative particle dynamics. J. Colloid Interf. Sci..

[37] Guo Z.Y., Ma L.P., Dai Q.X., Ao R., Liu H.P., Yang J. (2020). Combined application of modified corn-core powder and sludgebased biochar for sewage sludge pretreatment: Dewatering performance and dissipative particle dynamics simulation. Environ. Pollut..

[38] Groot R.D., Warren P.B. (1997). Dissipative particle dynamics: Bridging the gap between atomistic and mesoscopic simulation. J. Chem. Phys..

[39] Li Y., Leng M.T., Cai M.T., Huang L., Chen Y.W., Luo X.L. (2017). pH responsive micelles based on copolymers mPEG-PCL-PDEA: The relationship between composition and properties. Colloid Surface B.

[40] (2013). Materials Studio 7.0.

[41] Qiu B.N., Zhou Y., Yin X.Q., Chen J.H., Yin Y.Z., Zhu L. (2017). Preparation of citral microcapsules through spray-dring and the stability of the micorcapsules. Sci. Technol. Food Ind..

[42] Zhang S., Chen J., Yin X.Q., Qiu B.N., Zhu L. (2017). Preparation of citral microcapsules and the effects of water adsorption on the structure of micorcapsules. Food Sci. Technol..

[43] Groot R.D., Rabone K.L. (2001). Mesoscopic simulation of cell membrane damage, morphology change, and rupture by nonionic surfactants. Biophys. J..

[44] Ramezani M., Shamsara J. (2016). Application of DPD in the design of polymeric nano-micelles as drug carriers. J. Mol. Graphics Modell..

[45] Zwanzig R. (1961). Memory Effects in Irreversible Thermodynamics. Phys Rev..

[46] Wang W.J., Anderson N.A., Travesset A., Vaknin D. (2012). Regulation of the Electric Charge in Phosphatidic Acid Domains. J. Phys. Chem. B.

